# 

**Published:** 1857-01

**Authors:** 


### INDEX TO VOLUME IV.

PAGE

AIMS of this Journal.............. 58
Antecedents, the Best............ 87
Architecture, Defective........... 107
Air we Breathe..........159, 180, 249
Agricultural Publications......... 265
Advertising Morals.......122, 287, 268

Bodily Carriage................. 8
Burying Alive Prevented......... 35
Bites of Insects................43, 51
Beard Wearing.................. 71
Bodily Endurance............... 93
Bible Teaching.........90, 110, 111
Benevolence, True.............. 126
Benton, Thomas H............... 146
Benedictine.................... 194
Breath Measurement............. 221
Bread......................103, 240
Baths and Bathing.............. 251
Bad Colds..................183, 255
Burns and Scalds............... 259
Butter, Rancid, Cured........... 271

Crime and Disease............... 5
Common Sense................... 19
Corns Cured.................32, 288
Children's Reading...........44, 142
Coffee......................63, 284
Cornaro and Cornelius........66, 144
Country Life.................... 72
Consumption......49, 77, 179, 232, 248
Christian Missions, no Failure..... 91
Civility Profitable................ 97
Corn Bread, how made........... 101
Church and Theatre............. 113
Constitutions Ruined............ 117
Croakers....................... 120
Children's Sleep, &c.........214, 151
Colds, Bad.................255, 183
Coal, Laying in................. 202
Clergymen...11, 183, 187, 229, 240, 255
City Milk...................... 241
Condensed Milk................. 242
Celibacy....................... 245
Cold Bathing................... 251
Child Murder................... 283
Cheapest Food.................. 283

Disease and Crime.............. 5

PAGE

Drinking at Meals.............. 115
Disease and Benevolence......... 125
Divining Rods.................. 139
Death Made Easy................ 143
Diarrhœa...................... 170
Dictionary, Webster's........... 267
Dentifrices..................... 274
Dissolute Men.................. 284

Eating, a Year's................ 33
Eating and Drinking............. 63
Erring, Sympathy for the......... 160
Eating Economically............. 233
Editors' Requisites.............. 239
Eyes, Care of................... 273
Exchanges..................... 278

Family Music................... 21
Flour, too White............69, 102
Fruits, Uses of.................. 170
Felons, How Made............... 194
Flannel, Wearing and Washing... 254
Flour, for Burns and Scalds....... 259
Furnace Heat................... 276

Gloves and Shoes Tight.......... 36
Grape Vines.................... 94
Growing Beautiful............... 57
Going to the South...........149, 206
Going West..................... 209
Glories, The Three.............. 263
Girls of New York.............. 269

Home Made Happy.............. 41
Horseback Exercise............. 80
Heart Disease................81, 250
Houses, Healthfulness of......... 106
Hair Washes................... 121
Health Experiences..........156, 189
Happiness, True................ 163
Hydrophobia................... 167
"Hub Me Shipmate,".........237, 272
Hall, Stephen. Obituary.......... 244
HALL'S JOURNAL, aims of......... 272
Hot Air Furnaces................ 276
Hunger, Philosophy of........... 277
Healthy Country................ 282
Healthy Recreations............. 286
Hearing Improved............... 290

PAGE

Insanity and Suicide........16, 21, 110
Insect Bites, cured..............43, 51
Inverted Toe Nails................ 97
Isms of the Times................. 111
Idiots............................ 156
Intussusception..............169, 285
Inhalation..............185, 232, 248
Inheritance, the Best............. 282
Intemperance...................... 284

Journal's Work........24, 46, 58, 123

Kentucky's Children..........103, 238
Knowledge, Circling............... 181

Life's Last Sight................. 47
Living in the Country............. 72
Living Long..................66, 157
Life Destroyers................... 168
Lungs Measured.................... 221
Life-Times........................ 286

Morning Walks..................... 30
Milk, Proper Use of...........64, 240
Mother, Watchful.................. 142
Marriage Relation................. 155
Mortality of Cities........162, 185, 212
Miasm and Malaria................. 217
Measurement of Lungs.............. 221
Mental Power...................... 238
Mills Thornton, A., D.D.......104, 238
Milk of Cities.................... 241
Material for Men.................. 260
Marriage, Want, and Crime......... 260
Model Man......................... 261
Miscellaneous..................... 291

Newspapers........................ 44
Near-Sighted...................... 74
National Hotel Disease............ 197
New York Park Room................ 212
New York Mortuary................. 218

Occupations, Healthfulness of..... 49
Out-Door Activities...........80, 180
Our Sunshine...................... 192
Our Country....................... 216

Patent Diaper..................... 289
Parental.......................... 50
Plant a Vine...................... 94
Pone of Bread..................... 101
Politeness........................ 158
Punning........................... 161
Pepper and its Uses............... 180

PAGE

Physician, True, The.............. 188
Parks of Cities................... 212
Patriarch's Letters.......193, 208, 262
Poor, How to Keep the............. 270
Patent Medicines.................. 287

Rat Riddance..................51, 285
Reading Position.................. 178
Rust out, Wear out................ 192
Rail-Roading..............14, 48, 244
Recreations, Healthful............ 286

Suicide........................... 16
Supporters, Bodily................ 35
Snake Bites...................43, 51
Scarlet Fever..................... 53
Spinal Disease.................... 81
Soap Suds......................... 121
Secret Remedies................... 122
Spiritualism..................37, 139
Sleep of Children................. 151
School Children..............161, 168
Servants and Housekeeping....132, 173
Sleeping.....................45, 217
Spirometry........................ 221
Scalds and Burns.................. 259
Second Childhood.................. 281
Special Notice.................... 291

Tooth Washes..............32, 158, 274
Tea and Coffee.................... 63
Travelling for Health............. 69
Toe Nails, Inverted............... 97
Theatres and Churches contrasted.. 129
Throat Ail, Causes................ 145
Trouble Kills................152, 165
True Teachings.................... 161
Thorburn's Letters........193, 208, 262
Tea Drinking..............63, 234, 275
Table of Age...................... 257
Temperance True................... 258
Traps............................. 263
Time Table........................ 264
Three Articles.................... 283

Vine Planting..................... 94
Vermin Riddance..............263, 285

Water Cure........................ 89
Waste and Want.................... 133
Wife Worth Having................. 154
Wish, the Last.................... 159
Warts, Cured...................... 277
Water, to Purify.................. 287
Where are They?................... 284

